# Serotonin 5-HT2A receptor expression is chronically decreased in the anterior cerebral cortex of male rats following repetitive low-level blast exposure

**DOI:** 10.3389/fneur.2025.1594335

**Published:** 2025-06-25

**Authors:** Rita De Gasperi, Georgina Perez Garcia, Miguel A. Gama Sosa, Gissel M. Perez, Rania Abutarboush, Usmah Kawoos, Patrick R. Hof, Carolyn W. Zhu, Stephen T. Ahlers, Gregory A. Elder

**Affiliations:** ^1^Research and Development Service, James J. Peters Department of Veterans Affairs Medical Center, Bronx, NY, United States; ^2^Department of Psychiatry, Icahn School of Medicine at Mount Sinai, New York, NY, United States; ^3^Department of Neurology, Icahn School of Medicine at Mount Sinai, New York, NY, United States; ^4^General Medical Research Service, James J. Peters Department of Veterans Affairs Medical Center, Bronx, NY, United States; ^5^Department of Neurotrauma, Naval Medical Research Command, Silver Spring, MD, United States; ^6^The Henry M. Jackson Foundation for the Advancement of Military Medicine Inc., Bethesda, MD, United States; ^7^Nash Family Department of Neuroscience and Friedman Brain Institute, Icahn School of Medicine at Mount Sinai, New York, NY, United States; ^8^Department of Geriatrics and Palliative Care, Icahn School of Medicine at Mount Sinai, New York, NY, United States; ^9^Mount Sinai Alzheimer’s Disease Research Center and Ronald M. Loeb Center for Alzheimer’s Disease, Icahn School of Medicine at Mount Sinai, New York, NY, United States; ^10^Neurology Service, James J. Peters Department of Veterans Affairs Medical Center, Bronx, NY, United States

**Keywords:** blast, serotonin 5-HT2A receptor, male rats, military Veterans, traumatic brain injury

## Abstract

**Introduction:**

Many Veterans who experienced blast-related traumatic brain injuries (TBIs) in Iraq and Afghanistan currently suffer from chronic cognitive and mental health problems that include depression and post-traumatic stress disorder (PTSD). Male rats exposed to repetitive low-level blast develop chronic cognitive and PTSD-related behavioral traits that are present for more than 1 year after exposure. Psychedelic agents alter cognition as well as mood and agents such as psilocybin have gained attention as possible treatments for the mental health disorders that affect Veterans. The best-known action of psilocybin’s metabolite psilocin is to stimulate the serotonin 2A receptor (5-HT2AR). The aim of this study was to determine whether 5-HT2AR levels are altered by blast exposure.

**Methods:**

5-HT2AR expression was examined by Western blot in 7 cohorts of rats exposed to low level repetitive blast collected from 2 weeks to 12 months after blast exposure. The analysis included three brain regions (anterior cerebral cortex, hippocampus and amygdala) that were chosen based on being relevant to fear learning and the biological basis of PTSD. Possible correlations between Western blot data and behavioral outcomes were evaluated.

**Results:**

5-HT2AR was chronically decreased in anterior cortex of blast-exposed rats in all cohorts except the one studied at 2 weeks after blast exposure. 5-HT2AR levels were variably affected in the other regions. 5-HT2AR expression correlated differently in blast and control rats in some behavioral parameters.

**Conclusion:**

These findings have implications for understanding the neurochemical basis of blast-induced cognitive and behavioral changes. They also suggest 5-HT2AR as a potential therapeutic target for treatment of PTSD-related symptoms that follow blast injury.

## Introduction

Traumatic brain injuries (TBIs) occur for various reasons, but some are relatively unique to military settings, with blast exposure being the most prominent of them all. For Service Members deployed in Iraq and Afghanistan, most TBIs were caused by exposure to improvised explosive devices (IEDs) (1). Many of these Veterans currently show chronic cognitive and mental health problems such as depression and post-traumatic stress disorder (PTSD) (1). More recently there has been increased awareness and concerns in the military of the potential long-term effects of subclinical blast exposure now referred to as military occupational blast exposure (2), that commonly occurs for many Service Members during training and operations.

We have shown that rats exposed to repetitive, low-level blast develop chronic cognitive and PTSD-related behavioral traits (3–8) which appear several months after blast exposure and likely remain present for the animal’s lifetime (3, 4, 6). Since the condition these animals model is not induced by a psychological stressor as in PTSD, we have referred to it as blast-induced PTSD (5).

Treatments for TBI remain incompletely effective with many unsuccessful clinical trials (9). Treatment of PTSD where there is a history of TBI uses strategies similar to those used to treat PTSD in non-TBI settings. Strategies can be non-pharmacologic using trauma focused therapies or pharmacologic (10). For the latter, FDA approved selective serotonin reuptake inhibitors (SSRI) such as sertraline and paroxetine have shown some benefit (10). These drug act by increasing serotonin levels at the synapse via inhibition of the serotonin transporter (SERT). Serotonin-noradrenaline reuptake inhibitors (SNRIs) such as venlafaxine are also used. However, these treatments remain incompletely effective and better treatments are needed for Veterans with PTSD as well as TBI.

Serotonin 5-HT2A receptors (5-HT2AR) are a subtype of the serotonin receptor family found widely distributed in brain being particularly enriched in cortical layers I, IV and V mainly in pyramidal neurons (11). They are G protein coupled receptors (Gq/11), with primarily excitatory function. Abnormalities in 5-HT2AR signaling have been implicated in various mental health disorders including schizophrenia, depression and PTSD (12–14). In addition, 5-HT2AR signaling has roles in learning, memory and neurogenesis (15). The 5-HT2AR is also a major target for drug discovery in the treatment of serious mental health disorders (16, 17). Psychedelic agents alter mood, perception and cognition (18). Recently agents such as psilocybin have gained attention as possible treatments for PTSD (18). Psilocybin is a potent agonist of 5-HT2AR which is the best-known receptor involved in psilocybin’s mechanism of action (19).

There has been little study of the role of 5-HT2AR after TBI. In humans, 5-HT2AR autoantibodies in serum have been found to increase following TBI with the increases predicting declines in neurocognitive performance in older adult Veterans (20, 21). One study has examined 5-HT2AR expression after blast-induced TBI (22). This study showed that in mice exposed to a 20-psi closed-head blast exposure, 5-HT2AR ligand binding and receptor sensitivity increased in the immediate post injury period but there was no change in 5-HT2AR RNA expression at least at 10 days post injury (22).

Given the limited information regarding 5-HT2AR following blast injury and the fact that substances such as psilocybin seem promising for treatment of PTSD, we examined expression of 5-HT2AR in blast-exposed rats at various time points after exposure. Here we show that 5-HT2AR expression is chronically decreased after blast exposure. These findings have implications for understanding the neurochemical basis of blast-induced neurobehavioral traits. They also suggest that agents which target 5-HT2AR may treat the PTSD-related symptoms that follow blast injury.

## Materials and methods

### Animals

These studies used archived brain tissue from rats, that were blast or sham-exposed in prior studies (Table 1). Adult male Long Evans hooded rats (250–350 g; 10 weeks of age; Charles River Laboratories International, Wilmington, MA, United States) were used. All studies involving animals were approved by the Institutional Animal Care and Use Committees of the Walter Reed Army Institute of Research (WRAIR)/Naval Medical Research Command (NMRC) and the James J. Peters VA Medical Center. Studies were conducted in compliance with the Public Health Service policy on the humane care and use of laboratory animals, the NIH Guide for the Care and Use of Laboratory Animals, and all applicable Federal regulations governing the protection of animals in research.

**Table 1:** Summary of 5-HT2AR expression in all cohorts analyzed.

Cohort	References	Anterior cortex	Hippocampus	Amygdala
2 weeks	(33)	=	↓	↓
6 weeks (cohort 1)	(26)	↓	=	=
6 weeks (cohort 2)	(6, 30, 33)	↓	↓/=	↓
10 months (cohort 1)	(4, 33)	↓	=/↓	↓
10 months (cohort 2)	(33)	↓	=/↓	=
11 months	(31)	↓	↓	ND
12 months	(6, 30, 33)	↓	↑/=	=

=, no change; ND, not determined; ↑, increased expression; ↓, decreased expression; those indicated =/↓ or ↑/= depend on whether GAPDH or a Ponceau stain was used for normalization.

### Blast overpressure exposure

Exposure of rats to blast injury was performed using the WRAIR/NMRC shock tube that has been used in multiple prior studies to deliver blast overpressure injury to rats (3–7, 23–31). Anesthetized rats were randomly assigned to sham or blast conditions and placed in the shock tube in a prone position with the plane representing a line from the tail to the nose of the body in line with the longitudinal axis of the shock tube. The head was placed upstream, and its motion restricted during exposure to minimize rotational/acceleration injury. Further details of the blast exposure procedure and the physical characteristics of the blast wave are described in Ahlers et al. (32). Blast-exposed animals received 74.5 kPa exposures, equivalent to 10.8 psi, duration 4.8 ms, impulse 175.8 kPa*ms administered one exposure per day for three consecutive days. Control (sham exposed) animals were treated exactly as blast exposed animals except that they did not receive a blast exposure. Within 10 days after the last blast or sham exposure animals were transported in a climate-controlled van from the NMRC (Silver Spring MD, United States) to the James J. Peters VA Medical Center (Bronx, NY, United States).

### Animal housing

Animals were individually housed at a constant 70–72^o^ F temperature with rooms on a 12:12 h light cycle with lights on at 7 AM. Access to food and water was ad libitum. Subjects were housed on racks in random order to prevent rack position effects. Cages were coded to allow maintenance of blinding to groups during behavioral testing.

### Regional brain dissection

Animals were euthanized by CO_2_ inhalation. Regional brain dissections were performed as previously described (3). Tissues were flash frozen and stored at -80°C until used.

### Western blot analysis

Western blotting was performed as previously described (33). The antibodies used were a mouse monoclonal anti 5-HT2AR (SR-2A antibody (A4), sc-166775, Santa Cruz Biotechnology, Dallas TX, 1:600 dilution) and a rabbit monoclonal anti-GAPDH (Cell Signaling Technology, Danvers MA, #5174, clone D16H11, 1:2500 dilution) as loading control. Blot imaging was performed with Amersham 800 Imager (Cytiva, Marlborough MA) and quantified using Image Quant TL software (Cytiva). Blots were stained with Ponceau S solution (Sigma, St Louis) to obtain lane total protein load. 5-HT2AR expression was calculated as a ratio to GAPDH and to total protein load obtained from the integrated density of the Ponceau S-stained bands.

### Immunohistochemistry of rat brain

Immunohistochemistry was performed as previously described (34). Coronal sections (50-μm thickness) of 4% paraformaldehyde-fixed tissue from blast-exposed (6 weeks post-blast) and control animals were prepared with a VT1000S Vibratome (Leica Biosystems, Dear Park, IL). Antigen retrieval was performed by autoclaving the sections (15 psi, 120°C) for 10 min in 10 mM sodium citrate, pH 6.0 followed by slow cooling to room temperature. Sections were washed for 10 min in PBS (10 mM sodium phosphate, pH 7.2, 0.15 M NaCl), blocked for 1 h with 10% normal goat serum in 50 mM Tris HCl, pH 7.6, 0.15 M NaCl, 0.3% Triton-X-100 and incubated overnight with a mouse monoclonal antibody against 5-HT2AR (SR-2A, Santa Cruz Biotechnology) diluted 1:100 in blocking solution at room temperature. After washing with PBS (6 times for 10 min each), sections were incubated with anti-mouse IgG Alexa Fluor 488-conjugated secondary antibodies (1:300, ThermoFisher) in blocking solution for 2 h. After washing with PBS (6 times for 10 min each), the sections were mounted with Fluoro-Gel mounting medium (Electron Microscopy Sciences, Hatfield, PA). To visualize nuclei, sections were incubated in 0.1 μg/mL DAPI (4′,6-diamidine-2′-phenylindole dihydrochloride) in PBS in the next-to-last PBS wash. Sections were imaged with a laser scanning confocal microscope Zeiss LSM 980 with Airyscan 2 (Carl Zeiss Microscopy, White Plains NY).

### Statistical analysis

Values are expressed as mean ± SEM. Comparisons were performed using two-tailed unpaired *t*-tests or analysis of variance (ANOVA) followed by Tukey post-hoc tests. For all tests, statistical significance was set at a level of 0.05. Statistical tests were performed using the program GraphPad Prism 10.4.1 (GraphPad Software, San Diego CA). 5-HT2AR levels were correlated with behavioral measures previously reported in the same blast-exposed and control rats. Pearson’s product–moment correlation coefficients (*r*) and Spearman’s rank correlation coefficients (*p*) were calculated. *p*-values for differences between the slopes of blast and control groups were calculated using the Pearson *r* values by the method of Fisher (35, 36).

## Results

### Blast exposure reduces 5-HT2AR expression in anterior cerebral cortex

We examined 5-HT2AR expression by Western blotting in 7 cohorts collected in prior studies at various time points after blast exposure (Table 1). We focused on three anatomic regions (anterior cerebral cortex, hippocampus and amygdala) that were chosen because of their relevance to fear learning (37) and the biological basis of PTSD (38, 39).

**Figure 1:**
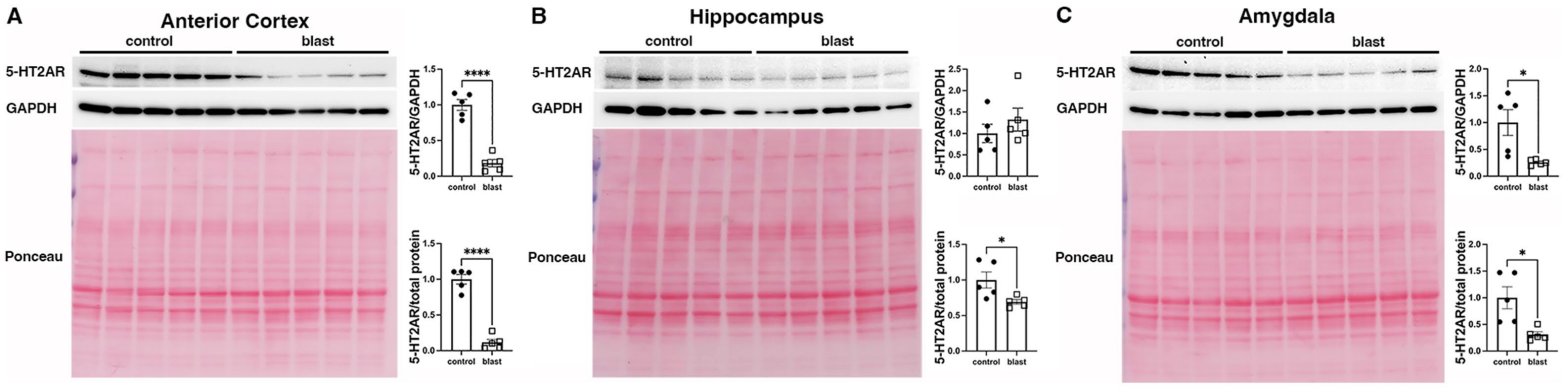
5-HT2AR expression in blast-exposed rats at 10 months following injury. 5-HT2AR expression was analyzed by Western blotting in anterior cerebral cortex **(A)**, hippocampus **(B)**, and amygdala **(C)** at 10 months after blast exposure (*n* = 5 control, 5 blast) (10 months post blast cohort 1 in Table 1). In each panel the top lane shows the 5-HT2AR blot, the middle the GAPDH blot and the bottom the Ponceau S-stained membrane. Bar graphs indicate 5-HT2AR levels expressed as a ratio to GAPDH or to total protein calculated from the Ponceau stain. Error bars indicate the standard error of the mean (SEM). Asterisks indicate values significantly different from controls (*p < 0.05, ****p < 0.001, unpaired *t*-tests).

**Figure 2:**
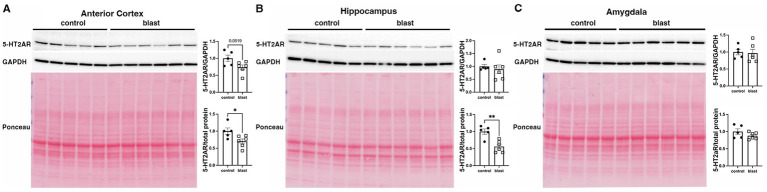
5-HT2AR expression in a second cohort of blast-exposed rats at 10 months following blast injury. 5-HT2AR expression was analyzed by Western blotting in anterior cerebral cortex **(A)**, hippocampus **(B)**, and amygdala **(C)** (*n* = 5 control, 6 blast) at 10 months after blast exposure (10 months post blast cohort 2 in Table 1). In each panel the top panel shows the 5-HT2AR blot, the middle the GAPDH blot and the bottom the Ponceau S-stained membrane. Bar graphs indicate 5-HT2AR levels expressed as a ratio to GAPDH or total protein calculated from the Ponceau stain. Error bars indicate the standard error of the mean (SEM). Asterisks indicate values significantly different from controls (*p < 0.05, unpaired *t*-test).

**Figure 3:**
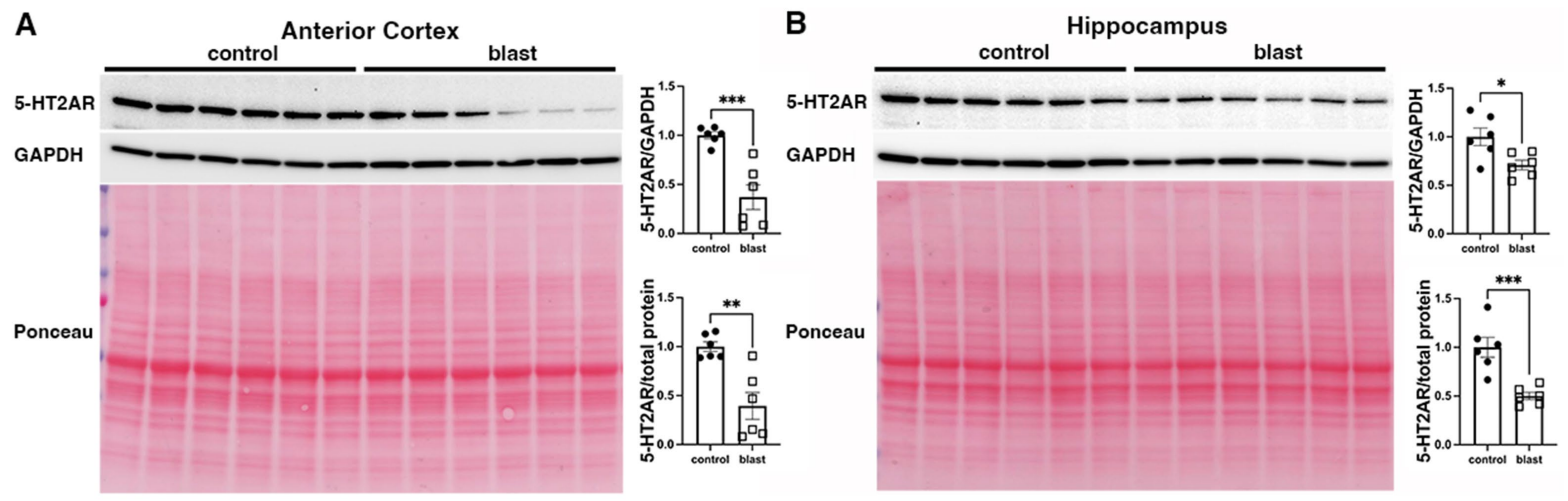
5-HT2AR expression in a cohort of blast-exposed rats at 11 months following blast injury. 5-HT2AR expression was analyzed by Western blotting in anterior cerebral cortex **(A)** and hippocampus **(B)** at 11 months after blast exposure (*n* = 6/group). The top image in the panels shows the 5-HT2AR blot, the second the GAPDH blot and the third the Ponceau S-stained membrane. Bar graphs indicate 5-HT2AR levels expressed as a ratio to GAPDH or to total protein calculated from the Ponceau stain. Error bars indicate the standard error of the mean (SEM). Asterisks indicate values significantly different from controls (*p < 0.05, **p < 0.01, ***p < 0.001, unpaired *t*-tests).

**Figure 4:**
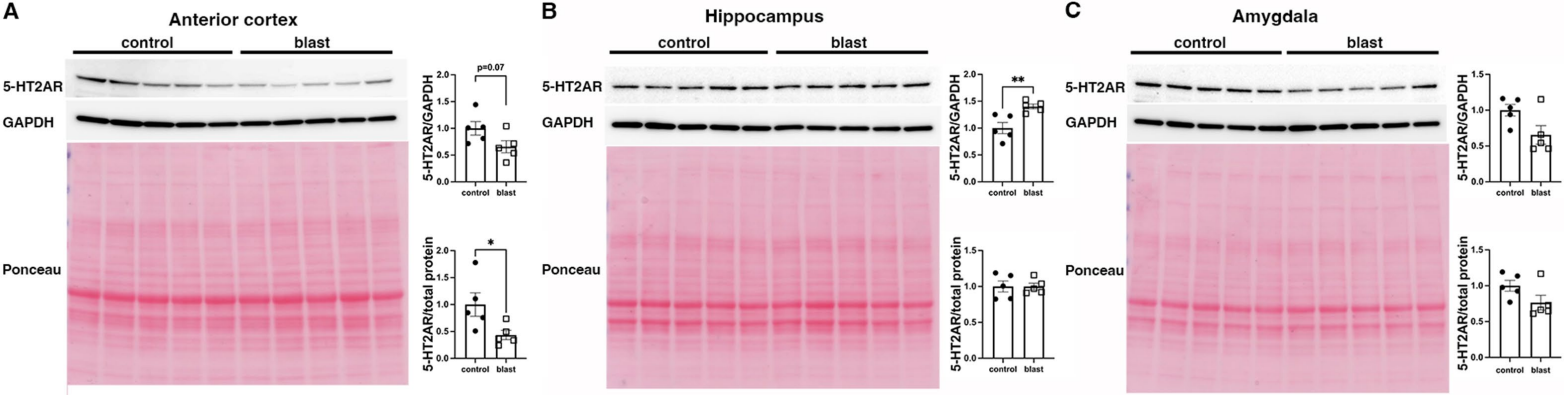
5-HT2AR expression in a cohort of blast-exposed rats examined at 12 months following blast injury. 5-HT2AR expression was analyzed by Western blotting in anterior cerebral cortex **(A)**, hippocampus **(B)**, and amygdala **(C)** at 12 months after blast exposure (*n* = 5/group). The top image in the panels shows the 5-HT2AR blot, the middle GAPDH blot and the bottom the Ponceau S-stained membrane. Bar graphs indicate levels expressed as a ratio to GAPDH or to total protein calculated from the Ponceau stain. Error bars indicate the standard error of the mean (SEM). Asterisks indicate values significantly different from controls (*p < 0.05, **p < 0.01, unpaired *t*-tests).

**Figure 5:**
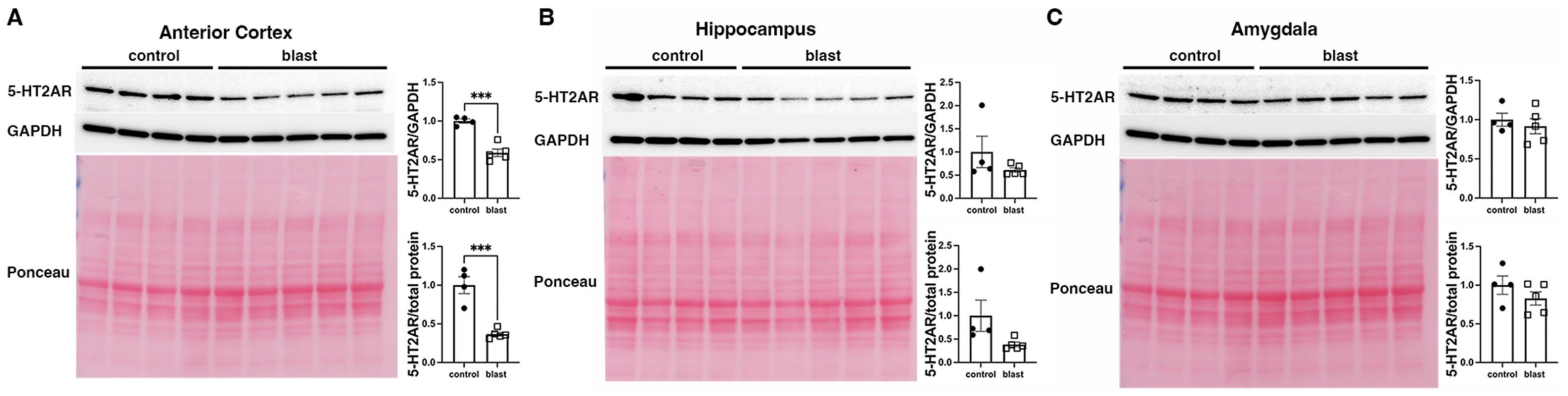
5-HT2AR expression at 6 weeks following blast injury. 5-HT2AR expression was analyzed by Western blotting in anterior cerebral cortex **(A)**, hippocampus **(B)**, and amygdala **(C)** at 6 weeks after blast exposure (6 weeks cohort 1 in Table 1) (*n* = 4 control and 5 blast). In each panel the top image shows the 5-HT2AR blot, the second GAPDH blot and the bottom the Ponceau S-stained membrane. Bar graphs indicate 5-HT2AR levels expressed as a ratio to GAPDH or to total protein load from the Ponceau stain. Error bars indicate the standard error of the mean (SEM). Asterisks indicate values significantly different from controls (***p < 0.001, unpaired *t*-tests).

**Figure 6:**
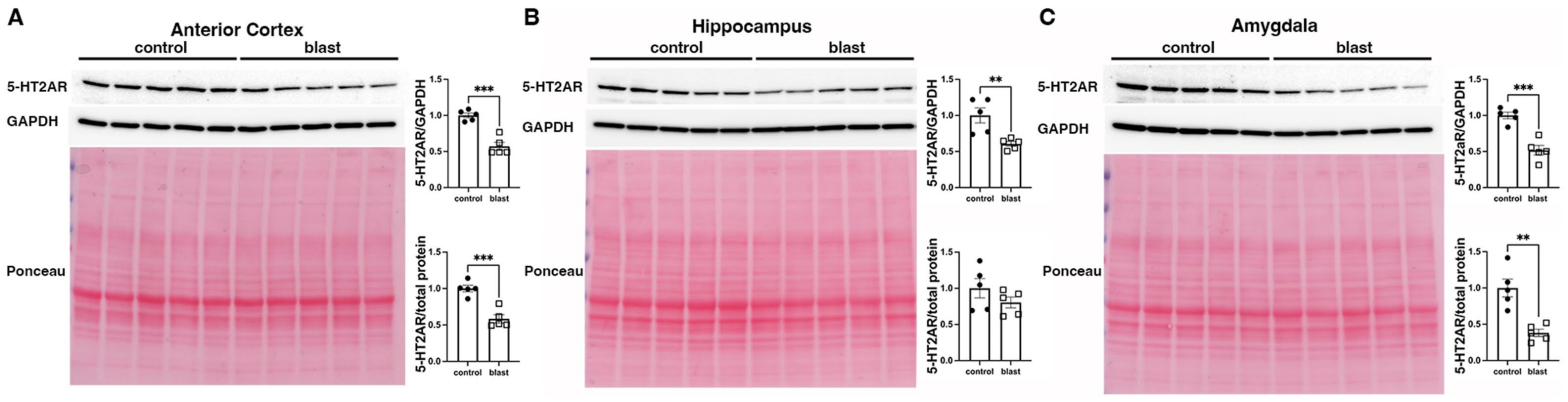
5-HT2AR expression in a second cohort of blast-exposed rats examined at 6 weeks following injury. 5-HT2AR expression was analyzed by Western blotting in anterior cerebral cortex **(A)**, hippocampus **(B)**, and amygdala **(C)** at 6 weeks after blast exposure (6 weeks cohort 2 in Table 1) (*n* = 5/group). In each panel the top image shows the 5-HT2AR blot, the middle the GAPDH blot and the bottom the Ponceau S-stained membrane. Bar graphs indicate 5-HT2AR levels expressed as a ratio to GAPDH or to total protein load from the Ponceau stain. Error bars indicate the standard error of the mean (SEM). Asterisks indicate values significantly different from controls (**p < 0.01, ***p < 0.001, unpaired *t*-tests).

**Figure 7:**
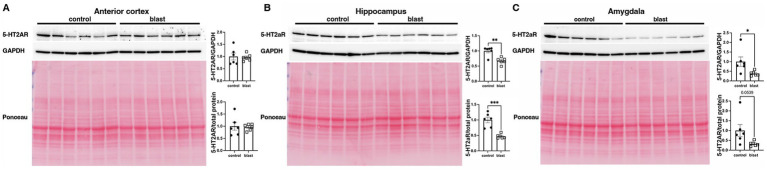
5-HT2AR expression at 2 weeks following blast injury. 5-HT2AR expression was analyzed by Western blotting in anterior cerebral cortex **(A)**, hippocampus **(B)**, and amygdala **(C)** at 2 weeks after blast exposure (*n* = 6/group). In each panel the top image shows the 5-HT2AR blot, the middle the GAPDH blot and the bottom the Ponceau S-stained membrane. Bar graphs indicate levels expressed as a ratio to GAPDH or to total protein from the Ponceau S stain. Error bars indicate the standard error of the mean (SEM). Asterisks indicate values significantly different from controls (*p < 0.05, **p < 0.01, ***p < 0.001, unpaired *t*-tests).

A summary of the results is presented in Table 1. In the initial cohort examined at 10 months (Figure 1) after blast exposure we found 5-HT2AR expression decreased in anterior cortex as well as amygdala but unchanged or decreased in hippocampus depending on whether the method of normalization was to GAPDH or to total protein load obtained from the integrated density of the Ponceau S-stained bands. In three additional cohorts examined between 10 and 12 months after blast exposure (Figures 2–4), 5-HT2AR was decreased in all three in anterior cortex while in amygdala and hippocampus, it was decreased, unchanged or in one case increased depending on the method of normalization (Table 1). To examine the time course of changes we analyzed two cohorts at 6 weeks after blast exposure (Figures 5, 6) and found in both that 5-HT2AR expression was decreased in the anterior cortex, while in hippocampus and amygdala 5-HT2AR was unchanged in one cohort (Figures 5B,C) and in the other decreased in amygdala and decreased or unchanged in hippocampus depending on the method of normalization (Figures 6B,C). When we examined 5-HT2AR at 2 weeks after blast exposure (Figure 7) we found it unchanged in anterior cortex (Figure 7A), while decreased in amygdala and hippocampus (Figures 7B,C). Thus across 7 cohorts of independently blast-exposed rats studied from 2 weeks to 12 months after blast exposure 5-HT2AR was decreased in the anterior cortex in all except the cohort studied at 2 weeks after blast exposure. By contrast, expression in hippocampus and amygdala was more variably affected.

**Figure 8:**
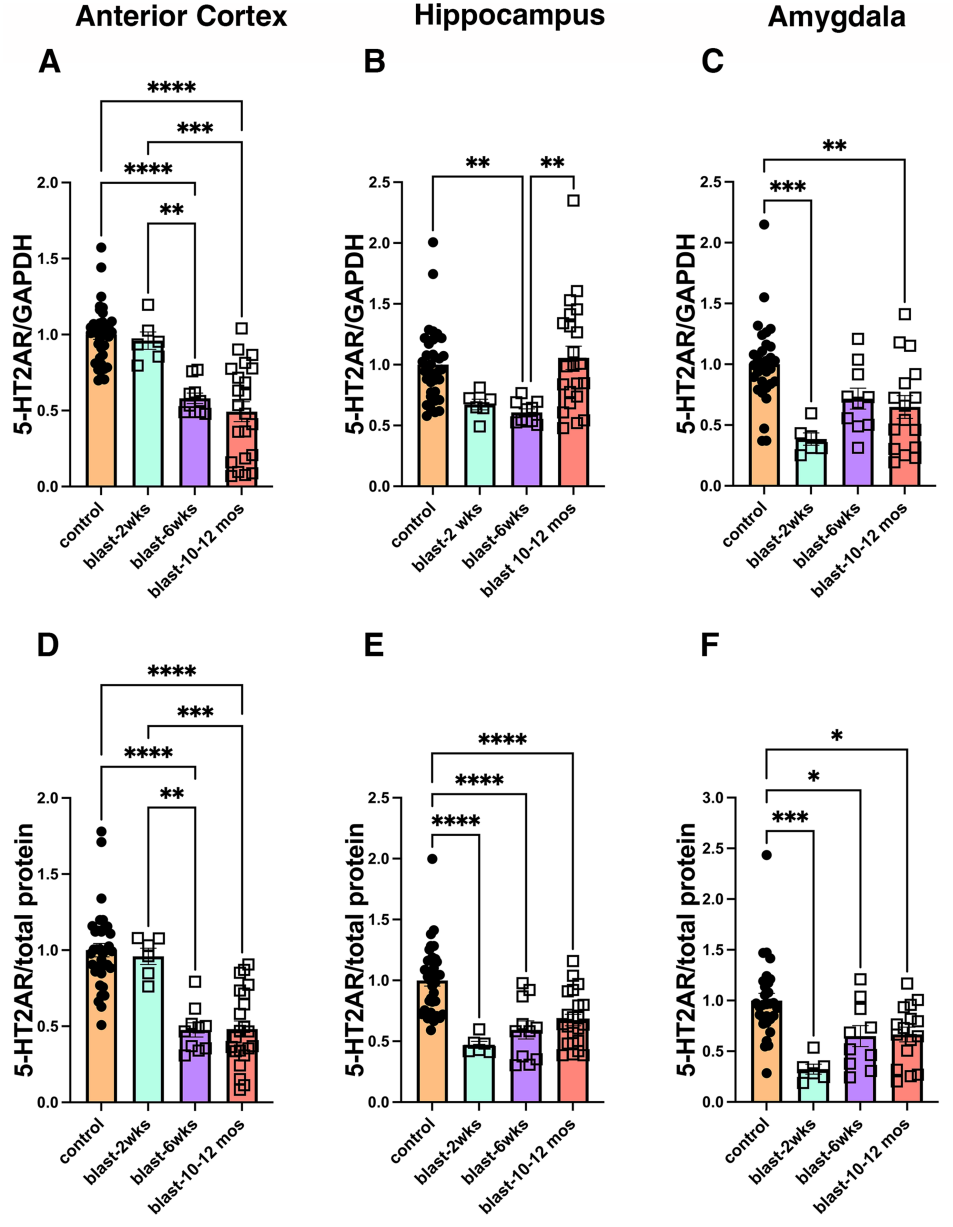
Comparison of 5-HT2AR expression in all cohorts across different time points (2 weeks, 6 months and 10–12 months). Bar graphs indicate levels expressed as a ratio to GAPDH or to total protein from the Ponceau S stain. For these studies all sham-exposed controls in Table 1 were pooled as well as blast-exposed animals at 10 and 12 months. **(A,D)**: anterior cerebral cortex, **(B,E)**: hippocampus, **(C,F)**: amygdala. Expression data were analyzed by one-way ANOVA (**A**: *F*_3, 70_ = 27.82, *p* < 0.0001, **B**: *F*_3, 70_ = 6.055, *p* = 0.0010, **C**: *F*_3, 58_ = 8.283, *p* = 0.0001, **D**: *F*_3, 69_ = 28.15, *p* < 0.0001, **E**: *F*_3, 71_ = 15.57, *p* < 0.0001, **F**: *F*_3, 58_ = 9.024, *p* < 0.0001). Asterisks indicate statically significant differences between groups (*p < 0.05, **p < 0.01, ***p < 0.001, ****p < 0.0001, Tukey post-hoc tests).

We also examined whether a correlation might exist between 5-HT2AR levels and time after blast exposure (Figure 8). For these studies since sham exposed controls at all time points were normalized to 1, we pooled all sham-exposed animals to create one control. We also pooled blast exposed animals between 10 and 12 months. In anterior cortex, 5-HT2AR levels steadily decreased over time while in hippocampus and amygdala 5-HT2AR levels were low at 2 weeks and remained decreased at later time points (Figure 8).

### 5-HT2AR immunostaining is reduced after blast exposure

**Figure 9:**
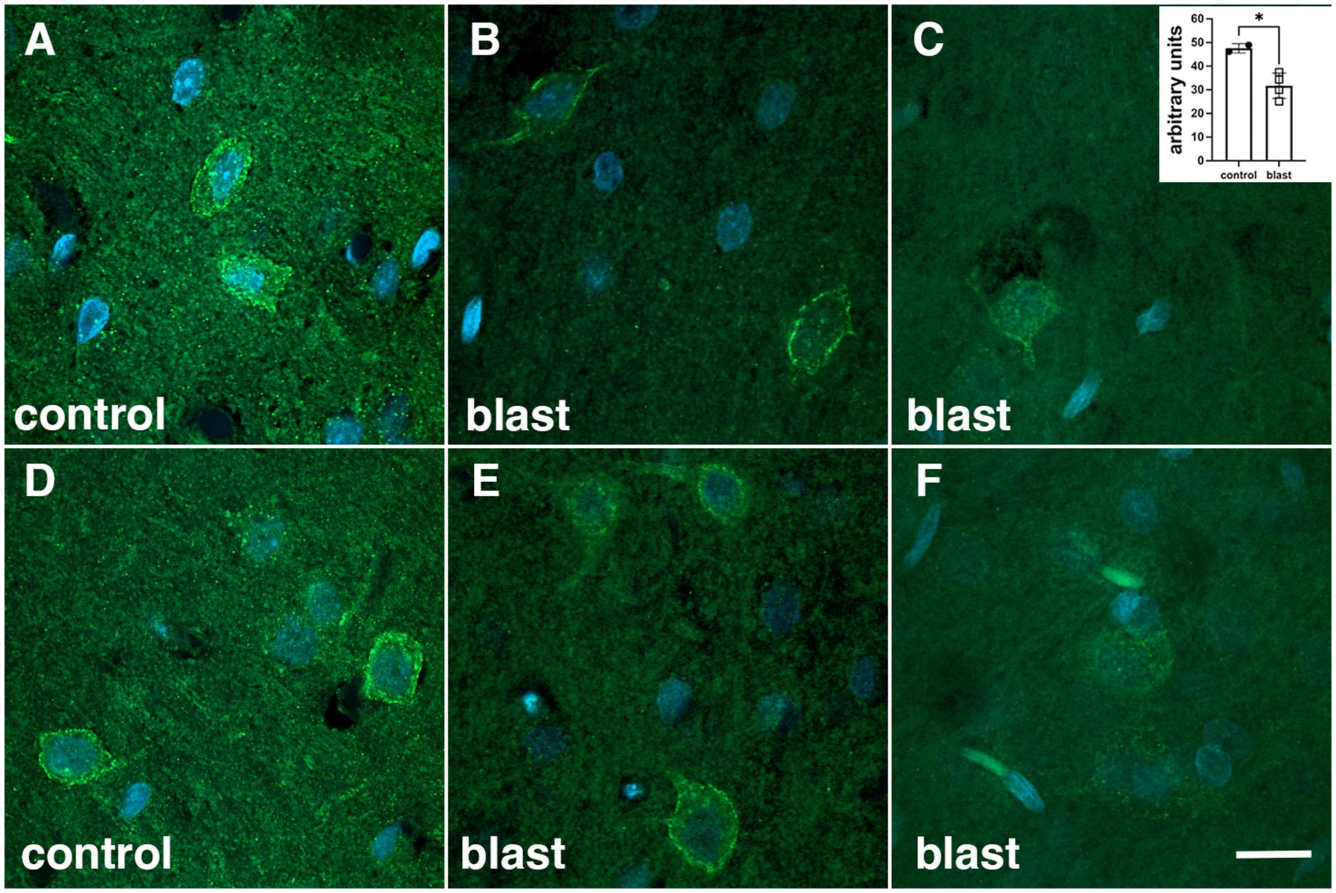
5-HT2AR expression in layer 4 of somatosensory cortex in control and blast-exposed rats studied at 6 weeks after exposure. Coronal sections were stained with the SR-2A monoclonal antibody against 5-HT2AR as described in Materials and Methods. Shown are two 0.15 μm optical sections of control **(A,D)**, and of two blast-exposed (**B** and **E**, **C** and **F**) animals. Insert in panel **(C)** shows quantitation of 5-HT2AR fluorescence in the panels (*p < 0.05, two tailed *t*-test). Scale bar 10 μm.

To examine whether the pattern of 5-HT2AR expression was altered following blast exposure, we examined by immunohistochemistry tissue sections from blast-exposed and control rats. Figure 9 shows immunohistochemical staining of layer 4 pyramidal cells within the somatosensory cortex at 6 weeks following blast exposure. Except for an apparent decrease in neuropil expression, there was no change in the overall pattern of expression.

### Correlation of 5-HT2AR levels with behavioral parameters

**Figure 10:**
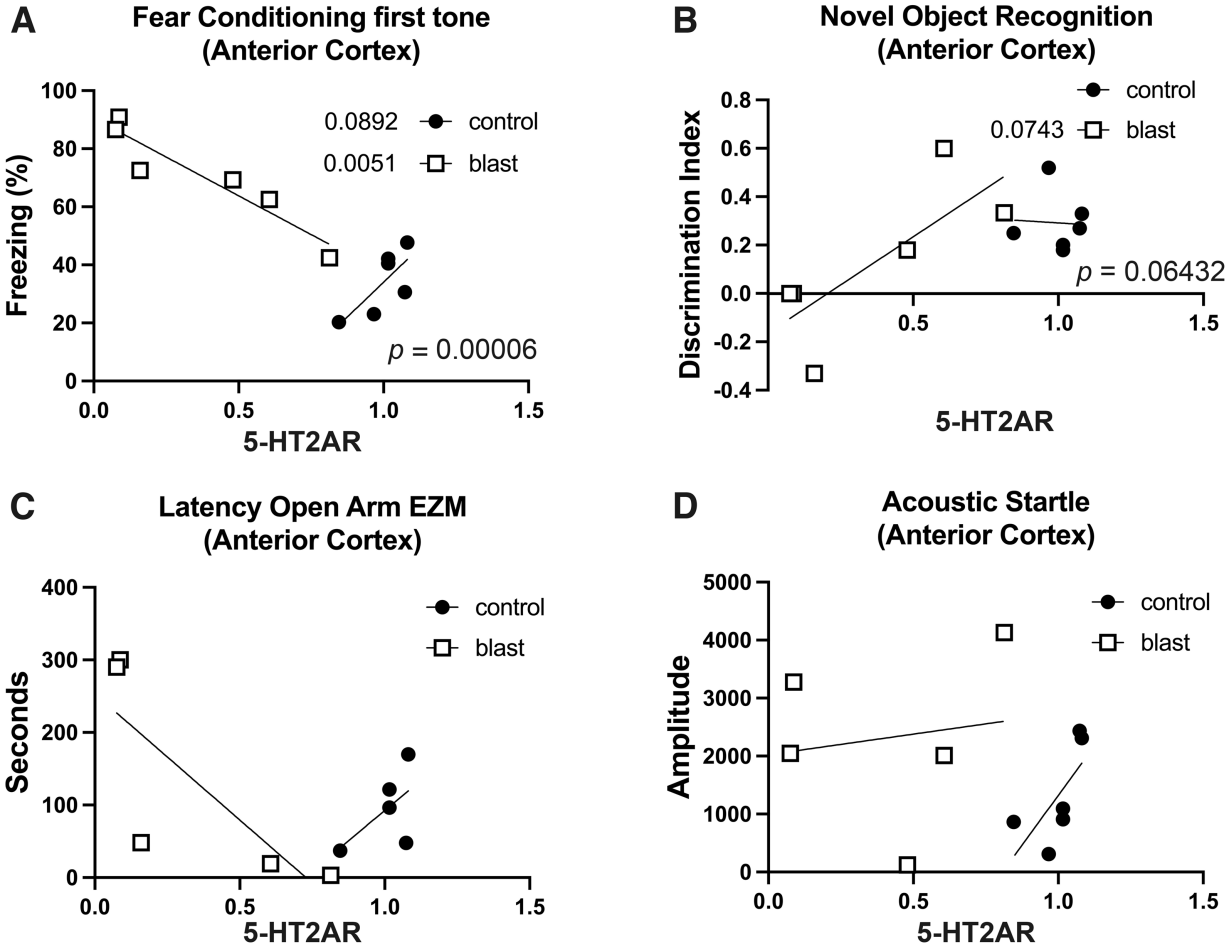
Correlation between 5-HT2AR expression and behavioral parameters. Shown is the correlation between 5-HT2AR expression and freezing to the first tone in the cued phase of a fear conditioning assay **(A)**, the discrimination index in the long-term memory testing of a novel object recognition test **(B)**, the latency to enter an open arm in an elevated zero maze (EZM; **C**) and the response to the first startle in an acoustic startle assay **(D)**. Data is derived from the 11-month cohort in Table 1. Pearson coefficients (*r*) are indicated. *p* values in the lower right of each panel indicate differences between the slopes of the blast and control lines calculated using the method of Fisher (35, 36).

In prior studies we have found it possible to correlate specific biochemical parameters with behavioral ones (28). We performed tests to determine whether 5-HT2AR expression might correlate with behavioral parameters. Figure 10 and Table 2 show 5-HT2AR expression correlated with blast-induced behavioral parameters in previously studied rats (11-month cohort Table 1) (31). Blast-exposed rats showed a strong negative correlation between 5-HT2AR levels and freezing to the first tone in the cued phase of a fear conditioning assay (*p* = 0.0051; Figure 10A) suggesting that lowered 5-HT2AR levels might be driving increased freezing in blast-exposed rats. By contrast in control rats, the slope of the line was positive although the *r* value did not reach statistical significance (*p* = 0.0895). However, the slopes of blast and control lines differed significantly (*p* = 0.00006). Similar trends were seen when comparing 5-HT2AR levels in the anterior cerebral cortex of this cohort with the discrimination index in a novel object recognition task (Figure 10B), latency to enter an open arm in an elevated zero maze (Figure 10C) and response to the first startle in an acoustic startle assay (Figure 10D). In this same cohort we did not find any significant correlations between behavioral parameters and 5-HT2AR expression in hippocampus (Table 2; tissue from amygdala was not available from this cohort for 5-HT2AR analysis).

**Table 2:** Correlations with behavioral parameters.

Region	Test	Control					Blast					Control and Blast
		Slope	Spearman *r* value	*p*-value correlation for Spearman	Pearson *r* value	*p*-value correlation for Pearson	Slope	Spearman *r* value	*p*-value correlation for Spearman	Pearson *r* value	*p*-value correlation for Pearson	*p*-value comparing slopes
11 months												
Anterior cortex	Fear C. Cued	95.79	0.7714	0.1028	0.7450	0.0892	−53.57	−0.9429	0.0167	−0.9413	0.0051	0.00006
Anterior cortex	NOR	−0.07526	0.08571	0.9194	−0.05254	0.9213	0.7683	0.7537	0.1111	0.7958	0.0743	0.06432
Anterior cortex	Zero M	−145.6	−0.08571	0.9194	−0.3228	0.5327	144.8	0.8286	0.0583	0.7191	0.1073	0.03236
Anterior cortex	SPI	−5,785	−0.3714	0.4972	−0.6533	0.1595	1,021	0.1429	0.8028	0.3402	0.5094	0.05000
Hippocampus	Fear C. Cued	17.20	0.2571	0.6583	0.4793	0.3361	−53.01	−0.8286	0.0583	−0.9000	0.0145	0.5619
Hippocampus	NOR DI	−0.005035	−0.4857	0.3556	−0.01245	0.9813	1.770	0.8208	0.1333	0.7765	0.1225	0.0703
Hippocampus	Zero M	−44.15	−0.02857	>0.9999	−0.3468	0.5006	79.52	0.7714	0.1028	0.3803	0.4571	0.1868
Hippocampus	SPI	−1,688	−0.1429	0.8028	−0.6754	0.1409	1921	0.3714	0.4972	0.6163	0.1926	0.07800
10 months (cohort 1)												
Anterior Cortex	Fear C. Cued	38.94	0.5000	0.4500	0.2489	0.6864	−42.46	0.1000	0.9500	−0.284	0.6434	0.3897
Anterior Cortex	NOR DI	−0.3067	−0.4000	0.5167	−0.2136	0.7302	1.677	0.9000	0.0833	0.7819	0.1182	0.04550
Anterior Cortex	Zero M	121.6	0.3000	0.6833	0.2964	0.6283	−438.4	−0.8000	0.3333	−0.7582	0.2418	0.04036
Amygdala	Fear C. Cued	−3.293	−0.1000	0.9500	−0.06727	0.9144	−310.3	−1.000	0.0167	−0.9117	0.0311	0.02034
Amygdala	NOR DI	0.3986	0.7000	0.2333	0.887	0.0448	2.043	0.2000	0.7833	0.4185	0.4831	0.1285
Amygdala	Zero M	38.81	0.6000	0.3500	0.3023	0.6211	−345.1	−0.4000	0.7500	−0.5198	0.4802	0.1615
Hippocampus	Fear C. Cued	−12.02	−0.3000	0.6833	−0.2164	0.7267	−8.670	−0.6000	0.3500	−0.2996	0.6243	0.8886
Hippocampus	NOR DI	−0.3686	−0.8000	0.3333	−0.9422	0.0578	0.2619	0.4000	0.5167	0.631	0.2537	0.00046
Hippocampus	Zero M	−62.52	−0.3000	0.6833	−0.4291	0.4709	−7.369	0.2000	0.9167	−0.03721	0.9628	0.5092
10 months (cohort 2)												
Anterior Cortex	NOR DI	0.3280	0.1539	0.8333	0.2540	0.6802	0.07474	0.6983	0.1667	0.05487	0.9178	0.74896
Anterior Cortex	Fear C. Cued	84.15	0.6000	0.3500	0.7822	0.1180	55.36	0.3143	0.5639	0.6930	0.1269	0.7564
Hippocampus	NOR DI	0.7818	0.2052	0.7333	0.4625	0.4329	−0.1083	−0.1518	0.8333	−0.1856	0.7247	0.28014
Hippocampus	Fear C. Cued	−82.95	−0.1000	0.9500	−0.5890	0.2960	28.11	0.8857	0.0333	−0.1856	0.0451	0.00374
Amygdala	NOR DI	−0.001172	0.2052	0.7333	−0.0007732	0.9990	0.2892	0.5161	0.3333	0.3302	0.5227	0.5892
Amygdala	Fear C. Cued	−71.30	−0.5000	0.4500	−0.5649	0.3211	2.621	−0.2571	0.6583	0.05103	0.9235	0.27572
12 Months												
Anterior cortex	NOR DI	−1.155	−0.2000	0.7833	−0.2523	0.6822	−0.1454	−0.2236	0.8000	−0.02936	0.9626	0.71884
Anterior cortex	Fear C. Cued	−369.0	−0.6000	0.3500	−0.7804	0.1194	139.8	0.7000	0.2333	0.2928	0.6325	0.03318
Anterior cortex	Zero Maze	414.5	0.7000	0.2333	0.4013	0.5031	89.06	−0.1000	0.9500	0.06391	0.9187	0.56868
Hippocampus	NOR DI	0.09308	0.1000	0.9500	0.1359	0.8275	1.191	0.7826	0.2000	0.7822	0.1180	0.14986
Hippocampus	Fear C. Cued	6.288	−0.2000	0.7833	0.08894	0.8869	−122.7	−0.7000	0.2333	−0.8360	0.0778	0.04036
Hippocampus	Zero Maze	−120.4	−0.9000	0.0833	−0.7795	0.1201	363.4	0.9000	0.0833	0.8477	0.0697	0.00046
Amygdala	NOR DI	0.4115	0.7000	0.2333	0.4665	0.4284	0.1231	−0.1118	>0.9999	0.2287	0.7113	0.6964
Amygdala	Fear C. Cued	−29.09	0.1000	0.9500	−0.3194	0.6003	−5.178	0.1000	0.9500	−0.09981	0.8731	0.7948
Amygdala	Zero Maze	20.30	0.3000	0.6833	0.3498	0.5638	9.679	−0.4000	0.5167	0.3050	0.6178	0.7788

Fear C, fear conditioning; NOR DI, novel object recognition discrimination index; values which are significant at 0.05 or less are indicated in red. Cohorts are those indicated in Table 1.

When we examined additional cohorts of rats, we found similar trends in one or more behavioral tests in all cohorts (Table 2) although it was difficult to associate any strong regional patterns with specific behavioral tests. Examining the slopes of the control and blast-exposed lines gave the most significant differences. In anterior cortex differences were found in novel object recognition and elevated zero maze in the 10-month cohort 1 and in fear conditioning in the 12-month cohort but no changes were seen in 10-month cohort 2. In hippocampus, slopes differed in novel object recognition (10-month, cohort 1) and fear conditioning (10-month, cohort 2) but not in other tests or cohorts. In amygdala, the only significant difference in slopes was for fear leaning in 10-month cohort 1. Clearly additional studies will be required with larger sample sizes and alternative methods to address the functional effects of receptor alterations, but these studies provide some support for the hypothesis that altered 5-HT2AR expression particularly in anterior cerebral cortex may be driving blast-induced behavioral traits.

## Discussion

We used a well-established animal model which employs male rats and mimics the type of open field low-level blast exposure associated with human mild TBI or subclinical blast exposure in humans (3–7, 23–31). The pressure level used (74.5 kPa, 10.8 psi) is high enough to be transmitted to the brain (40) but not sufficient to result in major gross neuropathological injury nor has systemic effects (32). The three exposures were given one each day for three consecutive days. While the number of three exposures was arbitrarily chosen it falls within the range of exposure that was commonly experienced by Service Members in Iraq (41, 42).

Male rats subjected to repetitive low-level blast exposure develop cognitive and PTSD-related behavioral traits that include anxiety, enhanced acoustic startle, and impaired recognition memory (3–6). The behavioral phenotype develops over time as it is not noted in the first 8 weeks after blast exposure but is consistently present at 3–4 months or later after exposure and remains present for over a year after exposure (3–7). Because of such features these animals represent a model to study the chronic neurobehavioral syndromes that often affect Veterans after blast exposure (43–45).

We show that 5-HT2AR expression is chronically decreased long after blast exposure. A summary of the results is presented in Table 1. Decreased 5-HT2AR expression in anterior cortex was found in all 4 cohorts examined at 10–12 months after blast exposure independent of the normalization method used (GAPDH or total protein), while in other regions 5-HT2AR expression was more variably affected. To examine the time course of changes we examined two cohorts at 6 weeks after blast exposure and found in both decreased 5-HT2AR expression in anterior cortex, while no change in anterior cortical expression was seen at 2 weeks after blast. Thus, across the 7 cohorts of blast-exposed rats studied from 2 weeks to 12 months after blast exposure 5-HT2AR was consistently decreased in anterior cortex except at 2 weeks, while its expression in hippocampus and amygdala was more variably affected.

One limitation of the study is that there is a large gap between the 6 week and 10-month time points examined. Future studies will be needed to examine 5-HT2AR expression during that critical time when the behavioral phenotype first appears. Another limitation of the study is the relatively modest sample sizes of the groups (*n* = 5–6). However, these sample sizes fall within a range in which we have in previous studies typically seen effect sizes of 1.8 or greater allowing an 80% power to detect 20% differences (21, 31).

Supporting a possible functional role, decreased 5-HT2AR expression correlated with some behavioral parameters. While no clear pattern emerged suggesting a regional correlation between decreased 5-HT2AR levels and changes in specific behavioral traits, these studies provide support for the hypothesis that altered 5-HT2AR expression particularly in anterior cerebral cortex may be driving blast-induced behavioral traits.

Why 5-HT2AR expression should be decreased after blast exposure is unclear. 5-HT2AR undergoes a complex cell type specific transcriptional regulation controlled by sequences in the 5′ flanking region of the gene (46). However, its major regulation occurs largely at the protein level during internalization and receptor recycling (15, 47, 48). Exploring the mechanisms by which blast affects 5-HT2AR levels will be an important next step in these studies.

The functional implications of decreased 5-HT2AR expression for the pathophysiology of blast-related brain injury is also unclear. However, it is noteworthy that it is observed well before the behavioral phenotype is present which only appears 3–4 months following blast exposure. Since decreases in 5-HT2AR levels in anterior cortex, correlate best with appearance of the behavioral phenotype (Figure 8), it is tempting to speculate that downregulation of 5-HT2AR in anterior cortex may somehow be involved in later development of the behavioral phenotype.

Rats exposed to repetitive low-level blast exposure also develop chronic elevation of the metabotropic glutamate receptor type 2 (mGluR2) in multiple brain regions (33). Interestingly, mGluR2 and 5-HT2AR affect several overlapping signaling pathways (15, 19, 47, 49, 50) and also have physical and functional interactions which suggest that the two receptors act as higher order receptor complexes (50–55). 5-HT2AR has been reported to be necessary for the agonist induced phosphorylation of mGluR2 at Ser843 (56).

Crosstalk between 5-HT2AR and mGluR2 appears to affect responses to antipsychotics and hallucinogens (55). The 5-HT2AR agonist-induced head-twitch response is absent in mGluR2 null mutant mice (53). Chronic treatment with the mGluR2/3 antagonist LY341495 decreases the head-twitch response to hallucinogens in wild type mice (54). Downstream signaling from the 5-HT2AR receptor through an HDAC2 histone modification affects mGluR2 gene expression (50). Exploring the interactions between mGluR2 and 5-HT2AR after blast exposure will be an important next step in understanding how blast exposure affects brain and behavior.

## Conclusion

The findings of this study have potential implications for treatment of the neurobehavioral syndromes that follow blast-induced TBI. Many drugs target 5-HT2AR (17, 57) with some demonstrating potential therapeutic benefit in PTSD and related disorders. For example, pimavanserin a selective 5-HT2AR inverse agonist which is in current clinical use as an antipsychotic for Parkinson’s disease (58), reversed multiple measures of anxiety in a rodent model of PTSD (59). Psychedelics such as psilocybin stimulate 5-HT2AR signaling (19). Psilocybin has recently received much attention as a possible therapeutic agent for many of the mental health disorders that affect Veterans including PTSD and depression (18, 60–62). Given the possible lowering of 5-HT2AR signaling following blast injury, future studies in this model with agents such psilocybin seem warranted.

## Data Availability

The original contributions presented in the study are included in the article/supplementary material, further inquiries can be directed to the corresponding author.

## Author disclaimer

The views expressed in this article reflect the results of research conducted by the authors and do not necessarily reflect the official policy or position of the of The Henry M. Jackson Foundation for the Advancement of Military Medicine, Inc., the Department of the Navy, or the Department of Defense (DoD). Mention of trade names, commercial products, or organizations does not imply endorsement by the U.S. Government. The study protocol was reviewed and approved by the Institutional Animal Care and Use committees of the Walter Reed Army Institute of Research/Naval Medical Research Command and the James J. Peters VA Medical Center. Studies were conducted in compliance with all applicable Federal regulations governing the protection of animals in research and in compliance with the Animal Welfare Act and per the principles set forth in the “Guide for Care and Use of Laboratory Animals,” Institute of Laboratory Animals Resources, National Research Council, National Academy Press, 2011. Some of the authors are military Service Members or federal/contracted employees of the United States Government. This work was prepared as part of their official duties. Title 17 U.S.C. § 105 provides that Copyright protection under this title is not available for any work of the United States Government. Title 17 U.S.C. § 101 defines a U.S. Government work as a work prepared by a military Service Member or employee of the U.S. Government as part of that person’s official duties.
